# Onyx Embolization of a Distal Middle Cerebral Artery Pseudoaneurysm in a Five-Week-Old: A Case Report and Review of Current Treatment Options

**DOI:** 10.7759/cureus.11974

**Published:** 2020-12-08

**Authors:** Yasmeen Elsawaf, Maryam Zeinomar, Andrea Scherer, Ravi H Gandhi

**Affiliations:** 1 Neurosurgery, University of Central Florida College of Medicine, Orlando, USA; 2 Pediatrics, University of Central Florida College of Medicine, Orlando, USA; 3 Pediatric Neurosurgery, Nemours Children's Hospital, Orlando, USA; 4 Neurosurgery, AdventHealth Orlando, Orlando, USA

**Keywords:** pseudoaneurysm, middle cerebral artery infarct, pediatric vascular malformation

## Abstract

Intracranial pseudoaneurysms secondary to traumatic birth are a rare finding in infants. Definitive diagnosis of such findings is challenging, and no standard management is delineated for management of pseudoaneurysms in the pediatric population. Commonly attempted treatments include endovascular embolization or surgical clipping. A 5-week-old female presented with a two day history of right hand focal seizures. The patient was found to have a dysplastic superficial intra-axial aneurysm arising from the distal left middle cerebral artery (MCA) branch in the setting of a left posterior frontal lobe hemorrhage noted on brain magnetic resonance imaging/magnetic resonance angiography (MRI/MRA). The patient underwent diagnostic cerebral angiogram demonstrating a left distal MCA pseudoaneurysm, which was treated with Onyx embolization. Post-embolization period was complicated by recurrent left central localized seizures and a left hemispheric temporoparietal hemorrhagic infarction. The patient was managed on levetiracetam, phenytoin, phenobarbital with stable seizure control. Herein, we highlight the youngest case to date of a 5-week-old infant with a left distal MCA pseudoaneurysm treated with Onyx embolization. Pseudoaneurysmal incidence, diagnosis and accepted management is discussed.

## Introduction

Intracranial pseudoaneurysms are exceedingly rare in the general population, consisting of less than 1% of diagnosed intracranial aneurysms [1-5]. Intracranial hemorrhage occurs secondary to pseudoaneurysm rupture in up to 60% of patients, with mortality ranging from 31-54% [6]. In the pediatric population, pseudoaneurysms represent 33% of all intracranial aneurysms [7]. In the current literature, there are 16 reported cases of middle cerebral artery (MCA) pseudoaneurysms in the pediatric population, three of which are in infants (5 weeks to 12 months of age) [1,8,9].

Pseudoaneurysms are defined by the lack of involvement of any of the three layers of the vessel wall (intima, media, and adventitia), as well as by the presence of organizing hematoma and fibrosis outside the true lumen [1]. As compared to true aneurysms, pseudoaneurysms with weaker supporting wall structure are highly unstable and prone to rupture [1,7]. The etiology of pseudoaneurysms in the pediatric population is most commonly secondary to trauma including motor vehicle collision, non-accidental trauma, or traumatic birth [1]. Decelerating forces from a motor vehicle collision or shaken-baby syndrome can cause shearing forces on the arteries, causing the vascular layers to weaken and yield the formation of traumatic pseudoaneurysms. Similarly, iatrogenic penetrating injury from the use of forceps or vacuum during birth or direct head injury can cause arterial injury with similar luminal compromise [10]. A less common cause of pseudoaneurysms may include collagen defects which yield intrinsic weakness in the arterial lumen; thus patients with genetic disorders such as Osteogenesis imperfecta, Ehlers-Danlos syndrome, and Marfan syndrome, as well as infectious diseases (endocarditis, arteritis, HIV) may present with pseudoaneurysms [1,11]. Idiopathic pseudoaneurysms have also been reported which may result from undiagnosed atherosclerosis in the adult population or underlying familial hypercholesterolemia, undiagnosed collagen defects, or unrecognized trauma in the pediatric population [12]. 

Diagnosis of intracranial pseudoaneurysms typically involves initial non-invasive imaging such as computed tomography (CT) or magnetic resonance imaging (MRI) with definitive diagnosis by a diagnostic cerebral angiogram. Differentiating an aneurysm from a pseudoaneurysm on CT or MRI is not possible due to the inability to define the vascular lumen. However, pseudoaneurysms are more commonly prone to rupture and must be suspected in the presence of underlying intraparenchymal hemorrhage with clinical evidence of recent trauma. A digital subtraction angiogram (DSA) allows for visualization of the false lumen. Current literature defines DSA findings of a pseudoaneurysm as an irregular vascular wall with a “snowman-like appearance.” Common angiographic findings in pseudoaneurysms include delayed lesion opacification, undefined aneurysmal necks, and delayed washout and venous phase retention of contrast [13].

Herein, we report the youngest case to date of a left MCA pseudoaneurysm in an infant that was treated with Onyx embolization. The incidence and diagnosis of intracranial pseudoaneurysms in the pediatric population are discussed, and the current literature on acceptable management strategies for this rare finding is reviewed.

## Case presentation

History and examination

A five-week-old female, who was born full-term (39 weeks gestation) via vacuum-assisted vaginal delivery with no complications, was noticed to have twitching in the right hand which persisted for 48 hours, with one episode of sustained right arm twitching for approximately five minutes. A head CT was obtained which demonstrated a left mid-parietal cortical hemorrhage with adjacent edema. 

**Figure 1:**
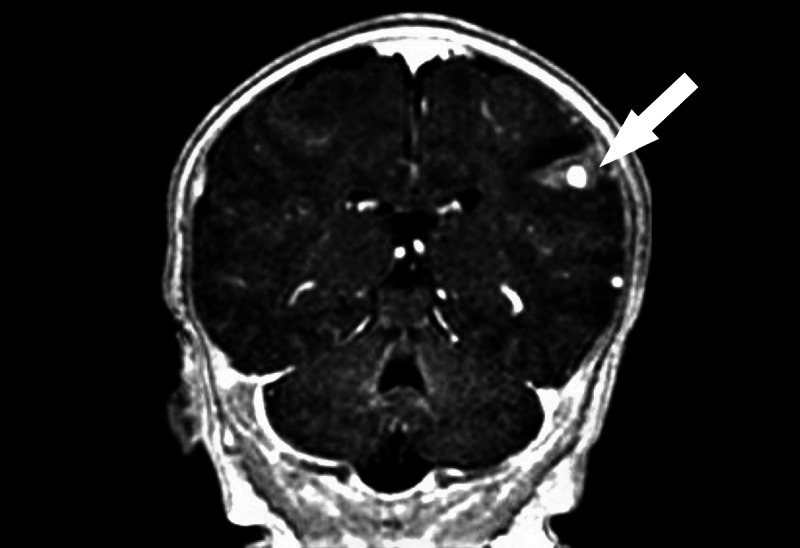
Pre-embolization MRI of the brain MRI of the brain depicting a dysplastic superficial intra-axial aneurysm arising at the distal left middle cerebral artery (MCA) branch with hemorrhage of the left posterior frontal lobe anterior to the central sulcus (white arrow)

An MRI of the brain suggested a dysplastic superficial intra-axial aneurysm arising at the distal left MCA branch in the setting of T1-weighted hyperintense, T2-weighted hypointense blood products in the left posterior frontal lobe anterior to the central sulcus (Figure 1); subsequent brain magnetic resonance venography (MRV) ruled out deep venous sinus thrombosis or flow-limiting stenosis. The patient was initiated on Levetiracetam 300mg and 24 hours later she underwent a diagnostic cerebral angiogram. Of note, a patent foramen ovale (PFO) was diagnosed on performing an echocardiogram during the hospital course.

Treatment

A diagnostic cerebral angiogram performed on the patient three days after symptom onset revealed a left distal MCA pseudoaneurysm which was embolized utilizing Onyx embolization.

Technique

**Figure 2:**
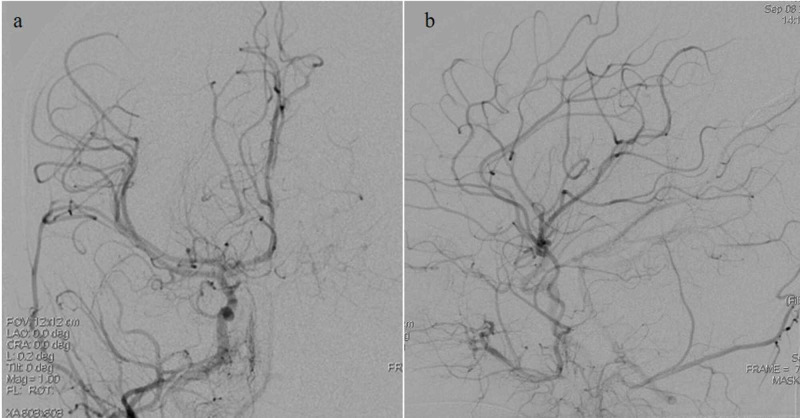
Pre-embolization digital subtraction angiography images obtained upon injection into the right internal carotid artery (ICA) a: right ICA injection demonstrating normal anatomy. b: left middle cerebral artery (MCA) pseudoaneurysm is not visualized.

**Figure 3:**
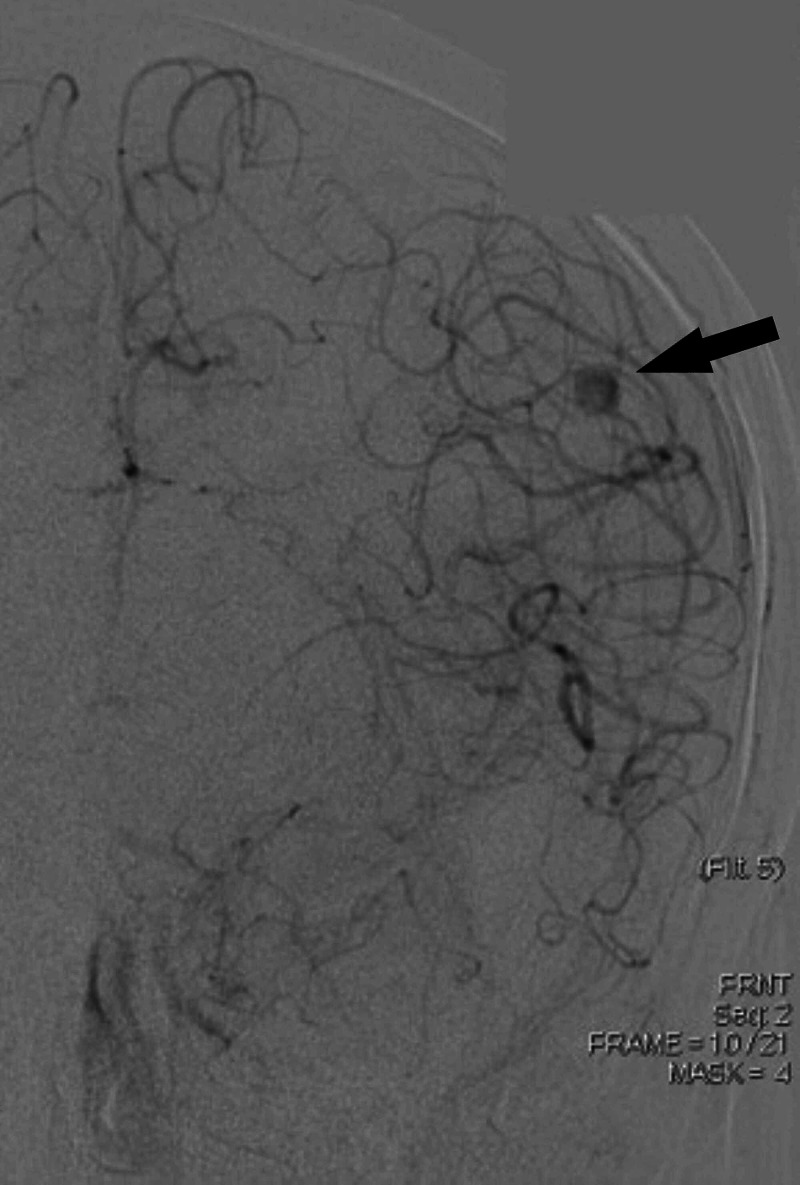
Pre-embolization digital subtraction angiography image obtained upon injection into the left internal carotid artery (ICA) Left ICA injection revealing the left middle cerebral artery (MCA) distal pseudoaneurysm (black arrow)

**Figure 4:**
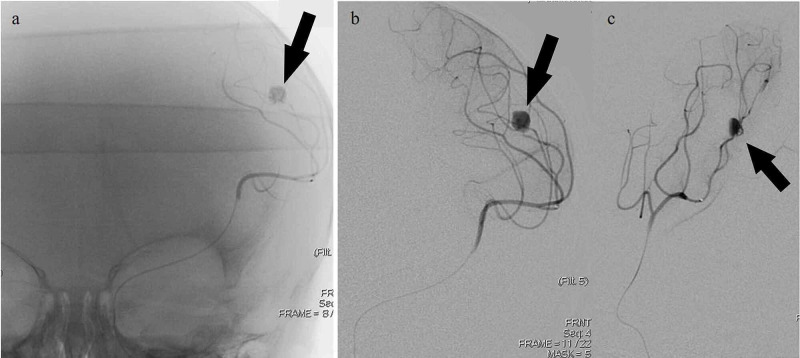
Pre-embolization digital subtraction angiography images obtained upon injection into the left middle cerebral artery (MCA); arterial phase a-c: Left MCA injection revealing the left MCA distal pseudoaneurysm (black arrow); arterial phase.

**Figure 5:**
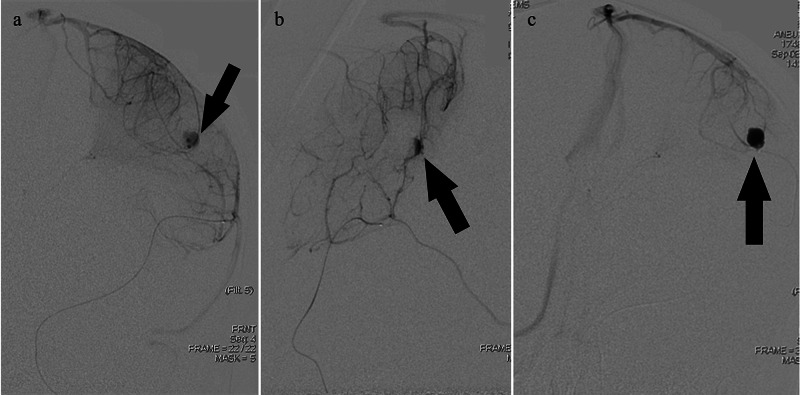
Pre-embolization digital subtraction angiography images obtained upon injection into the left middle cerebral artery (MCA); venous phase a-c: Left MCA injection revealing the left MCA distal pseudoaneurysm (black arrow); venous phase

The right femoral artery was accessed using a pediatric access kit and a 4-French sheath. A 4-French catheter was then used to select the right common carotid artery where the anteroposterior (AP) and lateral cranial runs were performed. Digital subtraction angiography of the right side (Figure 2) demonstrated the normalcy of the intracranial and extracranial branches, given the absence of pathologic intracranial filling or sources of hemorrhage. The left internal carotid artery was then selectively catheterized and AP and lateral cranial runs were performed. An aneurysmal dilatation arising at the posterior division of the MCA and M3 branch was noted as shown in Figure 3. Multiple obliques were obtained which demonstrated an artery entering and exiting the aneurysm and a slow filling consistent with a pseudoaneurysm. Digital subtraction angiography was used to visualize the left anterior circulation with a left distal MCA pseudoaneurysm in the arterial (Figure 4) and venous phases (Figure 5). There did not appear to be any early draining veins to suggest an arteriovenous malformation (AVM) or fistula.

**Figure 6:**
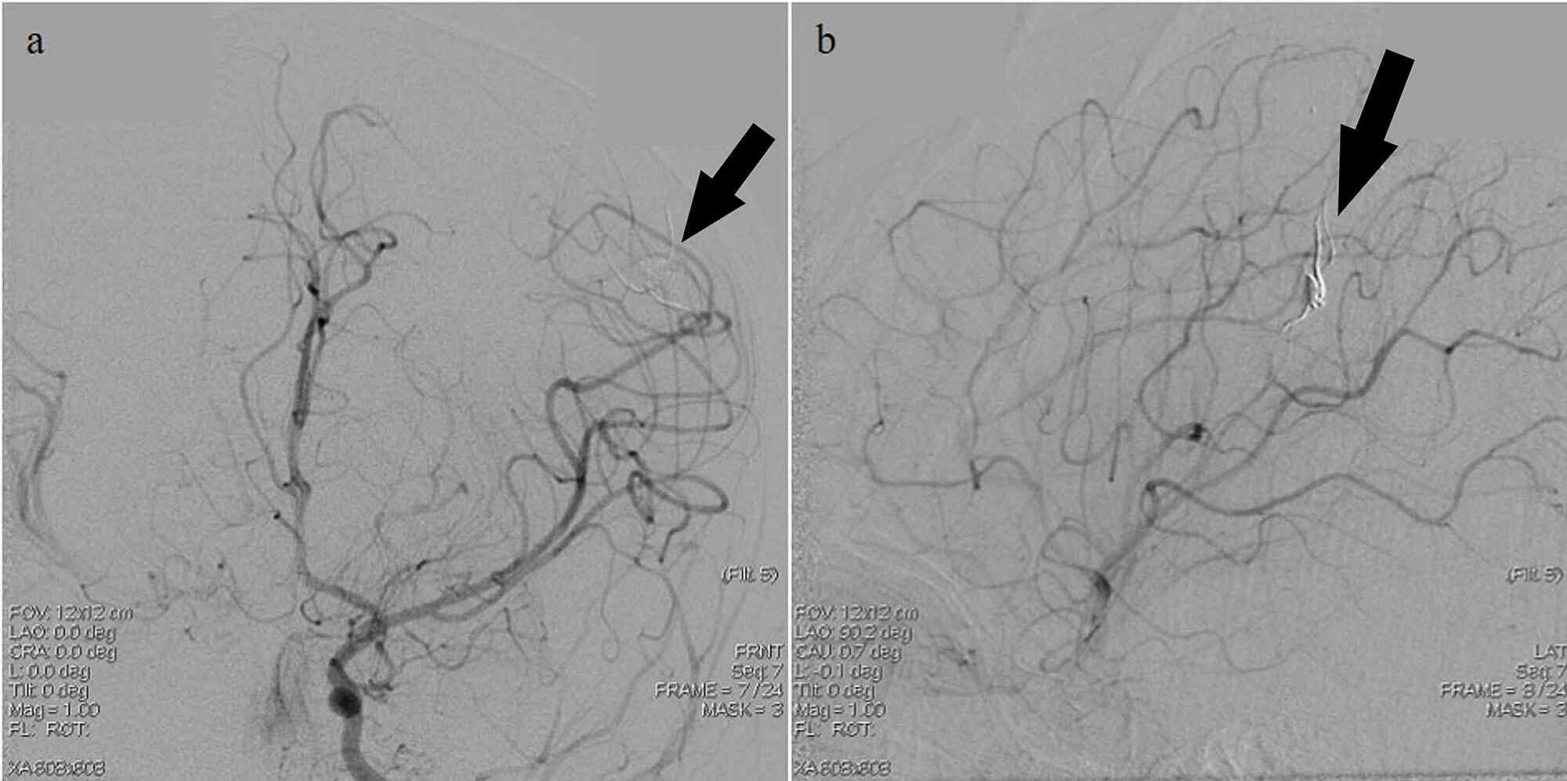
Post-embolization digital subtraction angiography images obtained upon injection into the left internal cerebral artery (ICA); arterial phase a, b: Left ICA injection revealing left middle cerebral artery (MCA) distal pseudoaneurysm (black arrow) in the arterial phase post-embolization

**Figure 7:**
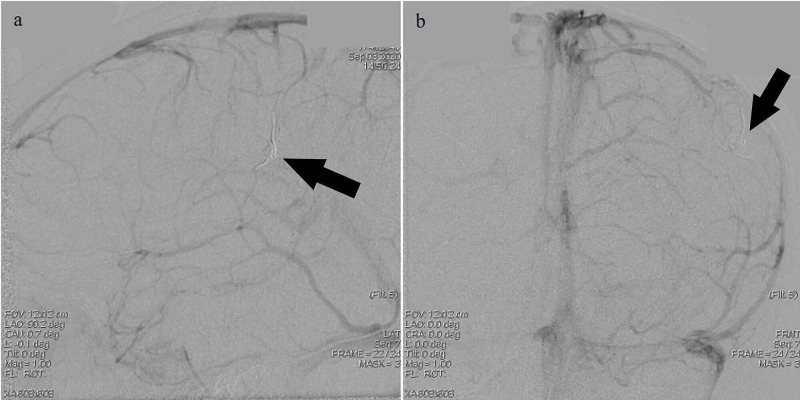
Post-embolization digital subtraction angiography images upon injection into the left internal carotid artery (ICA); venous phase a, b: Left ICA injection with visualization of the left middle cerebral artery (MCA) distal pseudoaneurysm (black arrow) in the venous phase post-embolization

A microcatheter was then advanced into the MCA up to the level of the aneurysm. Then dimethyl sulfoxide (DMSO) was injected through it, followed by Onyx 34 injection. The Onyx was used to fill the pseudoaneurysm. There was some Onyx reflux as well as some filling into the distal vessel to confirm that there would be no retrograde filling of the pseudoaneurysm. Digital subtraction angiography was performed again to visualize the left anterior circulation and the left distal MCA pseudoaneurysm post-Onyx embolization in the arterial (Figure 6) and venous (Figure 7) phases.

Postoperative imaging

MRI performed 24 hours post-embolization revealed a trace abnormal sulcal fluid-attenuated inversion recovery (FLAIR) signal in the region of the left frontoparietal lobe likely related to underlying subarachnoid hemorrhage. Abnormal restricted diffusion was noted involving the left temporal parietal lobe with the involvement of the perirolandic region and motor knob, extending to the region of the paracentral lobule. A small parenchymal hemorrhage (approximately 0.6 x 1.6 cm) with surrounding edema and superimposed subarachnoid hemorrhage were found to extend superiorly along the frontoparietal lobe. Sulcal effacement with abnormal hyperintense T2 weighted/ FLAIR signal was also noted within the area of the abnormal restricted diffusion.

Postoperative course

The postoperative course was complicated by left MCA infarct and status-epilepticus. Initially, twitching of the right hand persisted and the patient subsequently had one generalized seizure characterized by arching of the back and limb stiffness. Video electroencephalogram (EEG) monitoring noted frequent electrographic (consisting of rhythmic spikes and sharp waves) and clinical seizures originating from the left central region, associated with the MCA territory. One-week post-embolization, the patient was observed to be well controlled on levetiracetam 150 mg twice daily (60 mg/kg/day), phenobarbital 20 mg twice daily (8 mg/kg/day), and phenytoin 25 mg twice daily with the resolution of seizure-like activity. The patient was discharged and instructed to undergo an in-clinic follow-up with the neurology department for antiepileptic drug maintenance.

## Discussion

The largest case series of intracranial pseudoaneurysms in the current literature consists of 15 patients. In that series, six patients had pseudoaneurysms arising from the middle cerebral artery, all of which resulted from head trauma. Sixty-seven percent of those patients presented with a seizure [1]. In our case also, the patient presented with a seizure. This occurred due to the temporal lobe hemorrhage inducing meningeal irritation overlying the MCA distribution [14].

In the presented patient, the etiology of the left distal MCA pseudoaneurysm was suspected to be secondary to traumatic birth or neonatal injury. The patient underwent vacuum-assisted delivery which is a well-established risk for intracranial hemorrhage [15]. As previously mentioned, blunt trauma induces tears in the internal elastic lamina which yields dissecting pseudoaneurysms [16]. A traumatic origin for neonatal aneurysms is not well-established in the current literature, but few reports exist of infants over one month of age who presented with intraparenchymal hemorrhages secondary to ruptured aneurysms after a mechanically assisted traumatic delivery [16-18]. Additionally, the distal MCA segments superficially overlie the cerebral convexity, beneath the pterion, where significant molding of the skull vault can occur during delivery. Mechanical compressive, torsional, or distractive forces can disrupt the structural integrity of the arteries, and vacuum-assisted delivery provides an additional compressive force that may play a role in the pathogenesis of distal MCA pseudoaneurysms [16].

A less likely cause that should be considered is an underlying vascular luminal defect. Evidence for an underlying vascular luminal defect in the presented patient includes the noted PFO on the echocardiogram. PFOs may be associated with underlying collagen defects evidenced by the known presence of collagen-rich connective tissue at the fused foramen ovale [19]. Despite the scarcity of current literature on the topic, there may be an unknown association between collagen defects in those with PFOs and pseudoaneurysms.

Management of pseudoaneurysms in the pediatric population has significantly changed with the rise of endovascular embolization. Historically, surgical clipping and aneurysmectomy with primary repair of the parent vessel have been performed. Due to the lack of an intact vascular lumen and the presence of a true aneurysmal neck, surgical clipping is often unsuccessful and is associated with an increased risk of rupture [6]. Currently, endovascular embolization with coiling, or onyx embolization with or without flow-diversion, is the standard approach for treating intracranial aneurysms. Pseudoaneurysms present an additional challenge against definitive treatment with coil embolization due to the lack of an intact lumen to maintain the detachable coil material. The current rate of success for treatment of pseudoaneurysms with endovascular coil embolization was determined in a series of 11 patients 40% of whom showed recurrence during a two-month, follow-up angiogram (n=5) [20]. Thus, the standard initial approaches to pseudoaneurysms include one-staged treatment with diagnostic cerebral angiogram consisting of Onyx embolization with or without flow-diversion in the parent vessel. This management yields decreased morbidity and mortality as compared to open surgical approaches. A 0% rate of recurrence was reported following endovascular Onyx embolization in an eight-patient case series over a median follow-up of 1.5 years (range 0.4 to 2.6 years) [1]. In all endovascular modalities, the risk of thromboembolism versus hemorrhage must be weighed; however, a much greater risk of hemorrhage, infection, and prolonged recovery exists for surgical intervention. Thus, upon considering both safety and the recurrence rate, endovascular embolization is considered superior to surgical intervention for the treatment of pseudoaneurysms in the modern era.

## Conclusions

Herein, we discuss the youngest observed case to-date of a five-week-old infant who presented with two days of persistent focal seizures and was found to have a left distal middle cerebral artery pseudoaneurysm. Her diagnosis and management involved one-staged diagnostic cerebral angiogram with Onyx embolization. She required antiepileptic drugs post-operatively to obtain freedom from seizures.
